# Normative data for healthy elderly on the phonemic verbal fluency task – FAS

**DOI:** 10.1590/S1980-57642009DN30100011

**Published:** 2009

**Authors:** Thais Helena Machado, Helenice Charchat Fichman, Etelvina Lucas Santos, Viviane Amaral Carvalho, Patrícia Paes Fialho, Anne Marise Koenig, Conceição Santos Fernandes, Roberto Alves Lourenço, Emylucy Martins de Paiva Paradela, Paulo Caramelli

**Affiliations:** 1 Grupo de Pesquisa em Neurologia Cognitiva e do Comportamento, Departamento de Clínica Médica, Faculdade de Medicina da UFMG; Ambulatório de Neurologia Cognitiva do Hospital das Clínicas da UFMG, Belo Horizonte, MG, Brazil.; 2 Departamento de Psicologia, Pontíficia Universidade Católica do Rio de Janeiro, Rio de Janeiro, RJ, Brazil.; 3 Laboratório de Pesquisa em Envelhecimento Humano - GeronLab, Universidade do Estado do Rio de Janeiro, Rio de Janeiro, Brazil.; 4 Departamento de Neurologia, Universidade de São Paulo, São Paulo, SP, Brazil.; 5 Disciplina de Geriatria, Departamento de Medicina Interna, Faculdade de Ciências Médicas, UERJ, Rio de Janeiro, RJ, Brazil.; 6 CIPI – UNATI, Universidade do Estado do Rio de Janeiro, Rio de Janeiro, RJ, Brazil.

**Keywords:** healthy elderly, verbal fluency, educational status, normative data

## Abstract

Phonemic verbal fluency tests assess the production of words beginning with specific letters. Of these letters, the most frequently used are F, A and S. It is a sensitive test for assessing frontal lobe functions.

**Objective:**

To provide normative data for the elderly Brazilian population on the FAS test and to investigate the effects of age and schooling on test performance.

**Methods:**

The individuals were divided into three age groups (60–69, 70–79 and =80 years), and into four groups according to education (1–3, 4–7, 8–11 and 12 years). All subjects were assessed by the Mini Mental State Examination and the FAS. Data were analyzed with Student’s t test, ANOVA, simple linear regression and Spearman’s correlation.

**Results:**

We evaluated 345 cognitively healthy volunteers, 66.66% being female, aged 60 to 93 years, with an educational level ranging from one to 24 years. The average (number of items) ±SD for the whole sample was 28.28±11.53. No significant effect of gender was observed (*p=*0.5). Performance on the MMSE and education exerted a direct influence on FAS scores (*p*<0.001), with education being the most significant factor. A positive correlation was found between FAS and the MMSE (r=0.404; *p*<0.001).

**Conclusion:**

The performance of Brazilian elderly on the *phonemic verbal fluency tests-FAS* is significantly influenced by education, where individuals with higher educational level present better performance than those with fewer years of schooling. Age and gender did not prove significant with the FAS.

## Abstract (pt)

Testes de fluência verbal fonêmica avaliam a produção de palavras que se iniciam com determinadas letras. Destas, as mais utilizadas são F, A e S. É um teste sensível para avaliar funções do lobo frontal.

**Objetivo:**

Estabelecer dados normativos para a população idosa brasileira no teste de fluência verbal fonêmica FAS e investigar os efeitos da idade e escolaridade no mesmo teste.

**Materiais e Métodos:**

Os sujeitos foram divididos em três grupos de idade (60–69, 70–79 e =80 anos) e quatro de escolaridade (1–3, 4–7, 8–11, ≥12 anos). Todos foram avaliados pelo Mini-Exame do Estado Mental e fluência verbal fonêmica FAS. Os dados foram analisados com o test t de Student, ANOVA, regressão linear simples e correlação de Spearman.

**Resultados:**

Foram avaliados 345 idosos cognitivamente saudáveis, 66,66% do gênero feminino, idade entre 60 e 93 anos e escolaridade entre 01 e 24 anos. A média (número de itens) ±DP na amostra toda foi 28.28±11.53. Não houve influência do gênero nos resultados (*p=*0.5). A performance no MEEM e o nível educacional exerceram influência direta nos escores do FAS (*p*<0.001), com a educação sendo o fator mais importante. Uma correlação positiva foi encontrada entre os escores no FAS e MEEM (r=0.404; *p*<0.001).

**Conclusão:**

O desempenho de idosos brasileiros no *Teste de Fluência Verbal Fonêmica – FAS* é significativamente influenciado pelo grau de escolaridade, com os indivíduos com nível educacional mais elevado apresentando desempenho superior àqueles com menos tempo de estudo formal. Idade e gênero não apresentaram significância com o FAS.

The ageing process leads to anatomical and functional alterations that influence the capacity of an individual to adapt to the environment and also increase the incidence of illnesses. In Brazil, demographic data from the past two decades demonstrate a significant increase in life expectancy associated with a decrease in birth rate, similar to the pattern in developed countries over the last century.^1,2^ The high life expectancy causes an increase in the incidence of neurodegenerative illnesses and makes the differentiation between normal and pathologic ageing an important and challenging task for the clinician.^3^

The knowledge of alterations inherent to cognitive functioning for instance, is an important parameter to differentiate normal from pathological ageing. Therefore, it is essential to have robust knowledge on cognitive domains, as well as information on performance in specific tasks that assess these domains.^4^

Different cognitive domains are known to change during the normal ageing process, including executive functions. Some studies have shown changes in performance on executive tests,^5-8^ although there is still no consensus about the nature of such alterations, for instance whether they are primary or secondary to impairment in other cognitive abilities, such as memory^9,10^ or perceptual speed.^11^

The term “executive functioning” refers to those abilities that allow an individual to determine objectives, to formulate new and useful strategies to reach these goals and to follow, pursue and adapt according to the various circumstances that he/she might deal with, frequently during longs periods of time. The frontal lobes are considered responsible for the decisive aspects of these abilities.^12-17^

Several investigators^16,18,19^ have proposed procedures and instruments to assess executive functions, such as the Wisconsin Card Sorting Test (WCST), Stroop Test, Clock Drawing Test and Verbal Fluency.

There are two types of verbal fluency tasks: semantic and phonemic. Semantic fluency tests require subjects to say as many words as possible belonging to a certain category (e.g. animals, fruits) whereas phonemic fluency tasks require participants to say as many words as possible beginning with a specific letter, usually within one minute. It is a sensitive task for assessing frontal lobe functions, especially prefrontal left areas.^13,17,20-24^ Both tests require neither specific materials nor a long time to administer. The FAS test is probably the best known phonemic fluency test, and consists of saying words beginning with the letters F, A and S, one at a time for one minute.

The FAS test is related to the frequency of words beginning with each letter in each language. Considering that phonemes may differ between languages, the scores of phonemic verbal fluency are not comparable from one country to another.^22,23^ Moreover, in Brazil there is a marked heterogeneity in educational level, mainly within the elderly population.

Some studies have shown correlations for age, schooling and gender with performance in phonemic and category fluency tasks.^23,25-30^ Considering these factors, it is necessary to determine specific parameters for the Brazilian population, which should take into account the influence of the different demographic variables.

The goals of the present study are to provide normative data for the Brazilian population on the verbal fluency test – phonemic tasks – FAS – in healthy elderly, and to investigate the effects of age, schooling and gender on test performance.

## Methods

### Sample

The sample consisted of cognitively healthy elderly, who received outpatient care in university reference centers from Belo Horizonte, São Paulo and Rio de Janeiro.

All subjects were aged 60 years or more, who attained above education-adjusted scores on the Mini Mental State Examination^31,32^ (MMSE). The Brazilian version of the MMSE used was the one proposed by Brucki et al.,^33^ which was applied according to the specific instructions provided by these authors.

Additional inclusion criteria followed the recommendations from the *Mayo’s Older Americans Normative Studies*^34^ (MOANS): absence of active neurologic or psychiatric disease; absence of cognitive deficit; absence of use of psychotropic medication in quantities that can compromise cognitive functioning or suggest neuropsychiatric perturbation; independent life status; absence of previous history of disorders that could influence cognition.

The subjects were divided into three age groups (60–69, 70–79 and 80 or more years) and into four groups according to years of formal educational (1–3, 4–7, 8–11 and 12 or more years). Illiterate individuals were excluded.

### Procedure

The participants were instructed to generate as many words as possible beginning with letters “F’’, “A” and “S” within a 1-minute period for each letter, excluding proper nouns such as people’s, city and country names and the same word with a different suffix. The following instructions were given: “I will say a letter of the alphabet. Then, I want you to give me as many words you can that begin with this letter, as quickly as possible. For example, if I say B, you can say bed, big, but you can’t say proper nouns like Brazil or Beatriz. Also you can’t say the same word with a different ending”. Subsequently, the subjects were asked if they had understood these instructions.

Words with one, or more than one meaning were also considered, if the subject pointed out the alternative meaning. Words in other languages that were included in the Portuguese Dictionary and widespread words even if not in the dictionary also counted. When the participant corrected their response, this was not considered an error. The final score only included correct answers.

The following items were considered errors: intrusions (i.e.: when appropriate answers for a letter were given, but inappropriate in terms of letter used at that time; perseverations (i.e. same words were repeated twice or more); derivations (i.e.: words that varied in number, size, gender and verb conjugations).

This research was approved by the Ethics Committee from all three university centers and all participants signed the written informed consent.

### Statistical analysis

To verify the normality of sample, the Kolmogorov-Smirnov test (K-S) was used.

The Student t-test was used to investigate possible differences in the number of answers between male and female individuals. ANOVA analysis was employed for the comparison between the number of answers from the three age groups and the four groups according to educational level.

To verify possible associations between total FAS scores and the variables age, education, gender and MMSE scores, the data were adjusted by a simple linear regression model. Spearman’s correlation coefficient was used in exploring associations between MMSE and FAS scores.

All analyses were performed using the statistical software SPSS, version 15.0. The significance level considered was *p*<0.05.

## Results

The final sample was comprised 345 elderly individuals, 230 females and 115 males, aged 60 to 93 years (mean±SD=72.14±7.30 years) and with educational level ranging from 1 to 24 years (mean±SD=8.29±5.40 years).

The mean score on the FAS for the whole sample was 28.28±11.53. Table 1 shows the scores of the subjects on the MMSE, according to age and education. Table 2 depicts the FAS scores (mean and percentile distributions) according to age and education.

**Table 1:** MMSE scores for three age groups and four levels of education.

Education	60-69 yearsn=135					70-79 yearsn=160					80 years or moren=50			
	1-3	4-7	8-11	12 or more		1-3	4-7	8-11	12 or more		1-3	4-7	8-11	12 or more
	n=14	n=31	n=36	n=54		n=38	n=57	n=32	n=33		n=14	n=20	n=11	n=5
Mean	26.00	26.26	27.69	27.87		24.79	26.37	27.59	27.48		23.79	26.20	27.36	27.40
SD	2.48	1.80	1.28	1.06		2.15	1.83	1.07	1.17		1.52	1.82	1.12	0.54

**Table 2:** FAS scores and percentile distribution for three age groups and four levels of education.

Education	60-69 yearsn=135					70-79 yearsn=160					80 years or moren=50			
	1-3	4-7	8-11	12 or more		1-3	4-7	8-11	12 or more		1-3	4-7	8-11	12 or more
	n=14	n=31	n=36	n=54		n=38	n=57	n=32	n=33		n=14	n=20	n=11	n=5
Mean	18.29	26.13	31.92	38.72		18.32	25.95	28.75	32.03		20.64	26.15	28.91	34.00
SD	8.99	7.07	10.75	10.55		8.84	8.75	9.29	12.31		9.19	8.21	9.90	11.51
Median	16.50	28.00	31.50	36.50		16.00	26.00	28.00	32.00		18.50	26.50	28.00	37.00
Percentile														
5	5.00	14.00	17.00	22.00		6.00	13.00	16.00	12.00		7.00	15.00	18.00	22.00
25	13.00	20.00	23.50	31.00		13.00	20.00	21.00	26.00		16.00	19.00	20.00	22.00
75	24.00	31.00	38.00	47.00		24.00	31.00	34.00	38.00		26.00	32.00	33.00	42.00
95	33.00	35.00	49.00	61.00		35.00	43.00	46.00	60.00		45.00	41.50	49.00	47.00
99	33.00	40.00	63.00	63.00		40.00	46.00	47.00	63.00		45.00	44.00	49.00	47.00

**Graph 1:**
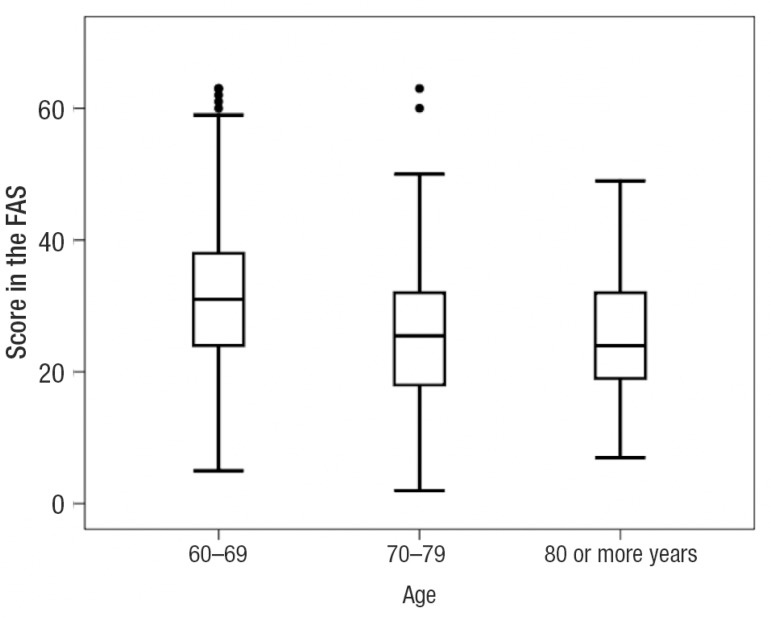
FAS scores with 95% CI in the three age groups.

**Graph 2:**
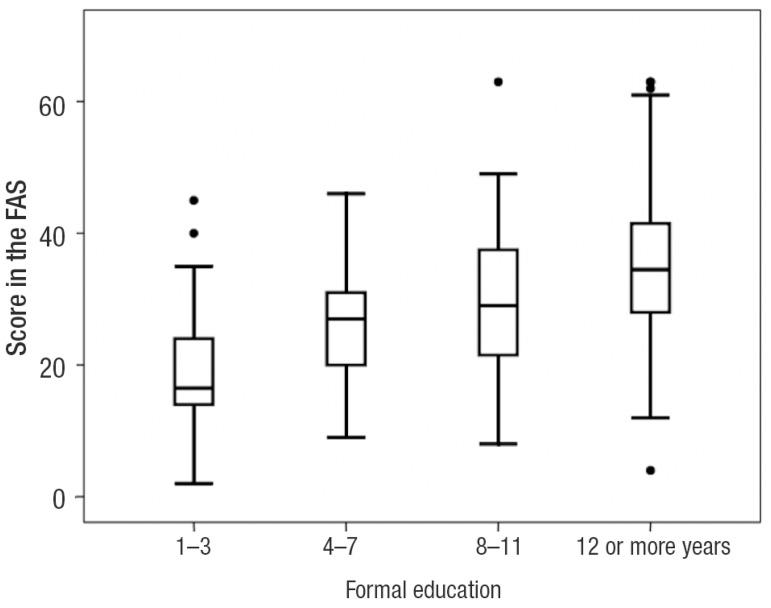
FAS scores with 95% CI in the four education groups.

Graphs 1 and 2 display the 95% Confidence Intervals (CI) related to age and education.

FAS scores for the overall sample presented a normal distribution (*p=*0.123). No significant difference was found between the number of FAS words produced by men and women (*p=*0.500).

The ANOVA analysis disclosed a correlation between certain age groups and schooling, albeit not a significant association. A simple linear regression model was calculated aiming to verify possible associations between FAS total score and the variables age, gender, education and MMSE scores (Tables 3 and 4).

**Table 3:** Results from the simple linear regression analysis obtained to verify the adjustment of the model.

Adjustment of model	
Statistics R	Variation explained
0.948	0.899

**Table 4:** Statistics obtained in the adjusted model for MMSE score and educational level.

Variable	Coefficient	*p* value
MMSE	0.599	<0.001
Educational level	4.810	<0.001

The variables selected in the adjusted model explain almost 89.9% of variation of FAS scores. The remaining 10.1% are probably related to other variables.

Performance on the MMSE and educational level exerted a direct influence on FAS scores, with education being the most important single factor. Moreover, a positive correlation was found between FAS and MMSE scores (r=0.404; *p=*0.000).

## Discussion

In this study, we presented normative values for the FAS verbal fluency test derived from a large sample of cognitively healthy individuals examined in three large cities from the southeastern region of Brazil. The population was stratified into three age groups and four levels of education in order to adequately investigate the effects of these variables on specific word production. FAS performance was significantly influenced by education, where subjects with higher schooling performed significantly better than their low schooling counterparts.

Although a trend toward an association between age and FAS performance was observed in one age group, this feature was not confirmed in the linear regression model. Similarly, no correlation between gender and test performance emerged. Other sociodemographic variables such as occupation and socio-economic level, which have not been taken into account in this study, could also influence test performance and may be related to the 10.1% variation in FAS scores not explained in the simple linear regression model.

The existence of normative data for neuropsychological tests in specific populations is highly important for it allows more precise diagnosis. This is of special relevance in countries where populations have marked heterogeneity of educational level, such as Brazil.

Some studies have shown correlations for age, schooling and gender with tasks of verbal fluency. Tombaugh et al.^23^ showed Canadian normative data stratified by levels of age and three levels of education for phonemic verbal fluency and also found a direct influence of education on this test. In regression analysis, education accounted for 18.6% of the variance, while age accounted for only 11.0%. Verhaeghen^35^ conducted a meta-analysis of studies on vocabulary, and disclosed that both age and education were found to be independent determinants of vocabulary performance. Dursun et al.^26^ investigated the effects of ageing and total years of education on verbal fluency test performance in healthy volunteers. Education and age were overall predictors of total FAS score, but no correlation with gender was found. Buriel et al.^27^ studied healthy young adults and found an influence of only education on FAS performance. Tallberg et al.^30^ provided normative data for the Swedish population on the FAS in 165 healthy individuals (16 to 89 years of age) stratified by education, age and gender. Educational level had a substantial influence on the performance in the test.

In Brazil, Brucki and Rocha^36^ analyzed the influence of education, gender and age on scores in a category fluency test (animals/minute) in 257 healthy adult individuals and concluded that education had the greatest effect on test results. Similarly, we have previously shown the need to use education-adjusted cut-off scores on the category fluency test for diagnosing Alzheimer’s disease in a sample of Brazilian elderly.^32^

The present study provided normative data for healthy elderly on the phonemic verbal fluency task – FAS, which was not hitherto available in Brazil. Performance is significantly influenced by education. We provided specific mean and percentile scores related to four different educational levels, which in the future may allow clinicians and researchers to use this test in the assessment of patients with cognitive impairment, as part of a diagnostic workup.
